# Emotional symptoms and cognitive function outcomes of subthalamic stimulation in Parkinson's disease depend on location of active contacts and the volume of tissue activated

**DOI:** 10.1111/cns.14187

**Published:** 2023-03-25

**Authors:** Kun Liang, Ren‐Peng Li, Yuan Gao, Chong Liu, Qiao Wang, Dong‐Mei Gao, Hui‐Min Wang, Liang‐Ying Zou, Xin Zhang, Chun‐Lei Han, Jian‐Guo Zhang, Fan‐Gang Meng

**Affiliations:** ^1^ Department of Functional Neurosurgery, Beijing Neurosurgical Institute Capital Medical University Beijing China; ^2^ Department of Functional Neurosurgery, Beijing Tiantan Hospital Capital Medical University Beijing China; ^3^ Beijing Key Laboratory of Neurostimulation Beijing China; ^4^ Chinese Institute for Brain Research, Beijing (CIBR) Beijing China

**Keywords:** cognitive function, deep brain stimulation, emotional symptoms, subthalamic nucleus, the volume of tissue activated

## Abstract

**Background:**

Subthalamic nucleus (STN) deep brain stimulation (DBS) is an effective treatment for Parkinson's disease (PD), that can improve patients' motor and non‐motor symptoms. However, there are differences in the improvement of patients' emotional symptoms and cognitive function.

**Objective:**

To investigate the impact of active contact location and the volume of tissue activated (VTA) on patients' emotional symptoms and cognitive function in STN‐DBS in PD.

**Methods:**

A total of 185 PD patients were included in this study. We evaluated them using the Movement Disorder Society‐Unified Parkinson's Disease Rating Scale, Hamilton Anxiety Scale (HAM‐A), Hamilton Depression Scale (HAM‐D), Montreal Cognitive Assessment (MoCA), and Mini‐Mental State Examination (MMSE) scales at the preoperative, 1‐ and 12‐month postoperative time points. Leads were positioned in standard space using the Lead‐DBS toolbox, and VTA was calculated for analysis.

**Results:**

When the lead active contact was closer to the ventral side of the STN, the patients' HAM‐A improvement rate was higher, and when the active contact was closer to the anterior and dorsal sides of the STN, the patients' MoCA improvement rate was higher. Stimulation of the sensorimotor zone was more favorable to the improvement of HAM‐A and HAM‐D in patients. And, the stimulation of the associative zone was more favorable to the improvement of MoCA in patients.

**Conclusion:**

Our results provide evidence that the 12‐month outcomes of cognitive function and emotional symptoms in PD patients with STN‐DBS were closely related to the specific location of the active contacts in the STN and influenced by the VTA.

## Graphical abstract

This study models the spatial distribution of activation contacts by correlating the improvement of patients' emotional and cognitive functions with the location of the spatial coordinates of the activation contacts. It was found that the outcomes of cognitive and emotional symptoms in PD patients with STN‐DBS were related to the specific location of active contacts in the STN and influenced by the VTA.

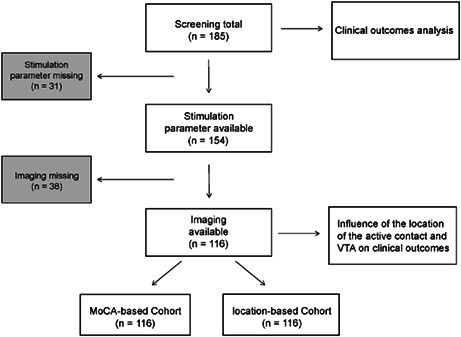

## 1 INTRODUCTION

In 1817, James Parkinson described a progressive neurological disease characterized by tremor, compulsion, and slowness of movements. Charcot later referred to the disease as Parkinson's disease (PD) in the 19th century.^1^ In 1987, Benabid et al.^2^ first described the application of deep brain stimulation (DBS) of targeting thalamic nucleus ventralis intermedius in order to treat PD patients at Grenoble university in France. Subsequently, subthalamic nucleus DBS (STN‐DBS) was confirmed to be effective to treat the symptoms of PD patients. It proved to be superior in controlling tremors, rigidity, and dyskinesia in patients with advanced stages of PD.^3^, ^4^

Although STN‐DBS has been proven to improve motor symptoms in PD patients,^5^, ^6^, ^7^ the effect of emotional symptoms and cognitive function has not been confirmed.^8^, ^9^, ^10^, ^11^, ^12^ In 2018, Kelley et al.^13^ experimentally confirmed that 4 Hz deep brain stimulation by STN improved cognitive performance, thus proposing that STN‐DBS may alleviate cognitive function in patients with PD. On the contrary, Drummond et al. summarized that verbal fluency, memory, planning, and cognitive flexibility consistently showed decline following STN‐DBS in 2020. These patients had increased decision thresholds when making rapid decisions, resulting in delayed responses, as well as more impulsive, incorrect choices during high‐conflict trials.^14^ On the other hand, in 2017, Enrici et al. suggested through their study that STN‐DBS may cause some psychiatric problems, such as depression and mania. However, they did not find a significant decrease in social cognitive function in patients through social–behavioral cognitive tests. Therefore, researchers currently believe that the effects of STN‐DBS on cognitive function, emotion, and behavior are still controversial.^15^

These studies neglected differences in location of active contacts and the volume of tissue activated (VTA). Therefore, we analyzed the data of STN‐DBS patients in our center and hypothesized that clinical outcomes of STN‐DBS in patients with PD depend on location of active contacts and the volumes of STN tissue, which was stimulated by the generated VTA (STN‐VTA).

## 2 METHODS

### 2.1 Patients and ethical approval

Parkinson's disease diagnosis was based on the MDS clinical diagnostic criteria 2015,^16^ and patients were selected for bilateral STN‐DBS according to guidelines of the International Parkinson and Movement Disorders Society from our center.^17^ Patients were excluded from the analysis when 12‐month follow‐up or pre‐/post‐operative imaging was missing. The research was carried out in accordance with the Declaration of Helsinki and authorized by local ethics committees (Beijing Tiantan Hospital, Capital Medical University, Beijing, China: KY2016‐037‐02). All patients gave written informed consent prior to study procedures.

### 2.2 Surgical procedures

All patients underwent standard frame‐based stereotaxic DBS surgery with bilateral STN (01/2018–12/2021). These patients received preoperative 3.0T MRI and high‐resolution CT examinations, and the Leksell–SurgiPlan (version 10.1) surgical system was used to fuse the images for visual localization of the bilateral STN targets. Intraoperative electrophysiological mapping with micro‐electrode recordings was performed to further determine the location of the implanted targets. Patients were given appropriate parameter adjustments 1 month after surgery to achieve maximum symptom improvement, and proper placement of stimulation lead was confirmed by high‐resolution CT.

### 2.3 Clinical assessment

Preoperative assessment was performed in the ON medication state as baseline status. Postoperative motor and emotional symptom evaluations were performed at 1 month (when the first stimulation was given) and 12 months in the ON medication and ON stimulation state. Postoperative cognitive function was performed only at 12 months in the ON medication and ON stimulation state. The following scales were used for clinical assessment:

1. Part III of the Movement Disorder Society‐Unified Parkinson's Disease Rating Scale (MDS‐UPDRS) was used to assess patients'motor symptoms.^18^
2. In terms of emotions, we focused on the patient's anxiety and depression symptoms. Hamilton Anxiety Scale (HAM‐A) and Hamilton Depression Scale (HAM‐D) were used to assess the emotional symptoms of patients.^19^
3. Patients' cognitive function was assessed using Mini‐Mental State Examination (MMSE)^20^ and Montreal Cognitive Assessment (MoCA) Beijing Version scales.^21^ In addition to the MoCA total score of the scale, we also focused on the score of each domain in the MoCA scale for patients, including Delayed free recall, Visuospatial and Executive abilities, Attention, Language, Orientation to time and place, and Name and Abstraction test.

### 2.4 Imaging data processing

We processed patients' imaging data using the Lead‐DBS toolbox (www.lead‐dbs.org) as described by Horn et al.^22^ First, preoperative MRI and postoperative CT were registered, and brainshift was corrected. Then, images were nonlinearly normalized into standard space (MNI ICBM 2009b NLIN, Asym) using advanced normalization tools. The pre‐reconstructed DBS leads were calculated using the PaCER or TRAC/CORE algorithm, and deviations were manually refined. The Lead‐Group was used to display 3D images of patients in the standard space. After that, the locations of activated contacts were displayed and the coordinates in the MNI space were counted. Finally, the VTA was calculated based on their stimulation parameters.^23^ All computational work was carried out with MATLAB 2022a.

### 2.5 Statistical analysis of clinical outcomes

All statistical analyses were performed using SPSS 22.0, R 4.1.3, and python 3.6. Scores are reported as mean ± standard deviation (SD) and the Shapiro–Wilk test was employed to check the normality of the data. Depending on the normality of the data, a paired *t*‐test or a Wilcoxon signed‐rank test was performed to find significant changes from baseline. For multiple comparisons, the *p*‐values were corrected by Bonferroni's test. The Friedman test was used for comparison of variables that did not obey a normal distribution at different time points before and after the intervention. The Kruskal–Wallis test was used for comparison between multiple groups. The relationship between the rate of improvement in MDS‐UPDRS‐III scores and the coordinates of VTA and lead activation contacts was explored by Pearson's correlation analysis. *p* < 0.05 was considered a statistically significant difference. The improvement rate of patients' motor symptoms and emotional symptoms was, respectively, calculated using the MDS‐UPDRS‐III scale and HAM‐A, HAM‐D ([(Test_baseline_ − Test_follow‐up_)/Test_baseline_] × 100%), and cognitive improvement rates were calculated using the MMSE, and MoCA scales ([(Test_follow‐up_ − Test_baseline_)/Test_baseline_] × 100%).

## 3 RESULTS

### 3.1 Patient characteristics and clinical outcomes

A total of 185 patients were included from the database, including 100 males and 85 females, with an age of 63.2 ± 8.15 years and a disease duration of 9.6 ± 5.62 years at the time of surgery. Amedian Hoehn and Yahr score was 3.0 (interquartile range from 2.0 to 3.0).

We found that motor symptoms, anxiety, and depression improved significantly after stimulation at the 1‐month follow‐up and the 12‐month follow‐up compared to baseline. At the 12‐month follow‐up, the patients' MoCA and MMSE scores did not change significantly from baseline, but an explorative analysis of MoCA domains showed a significant improvement in the Delayed free recall, which is shown in Table 1.

**TABLE 1:** Clinical outcomes.

Scale	(A) Baseline		(B) 1‐month follow‐up		(C) 12‐month follow‐up		*F*/*t*	*p*	Change
	Mean	SD	Mean	SD	Mean	SD			
MDS‐UPDRS‐III	47.22	22.23	26.19	18.02	25.17	15.55	94.218	<0.001	A > B* A > C* B > C
HAM‐A	16.86	9.85	11.87	7.40	12.16	8.77	15.35	<0.001	A > B* A > C* B > C
HAM‐D	17.11	9.29	11.94	7.77	13.44	10.49	12.92	<0.001	A > B* A > C* C > B
MoCA	20.65	4.96	—	—	21.22	5.69	−1.471	0.143	—
MoCA domains									
Visuospatial and Executive abilities	2.78	1.52	—	—	2.74	1.62	0.358	0.721	—
Name	2.64	0.74	—	—	2.66	0.77	−0.171	0.864	—
Attention	4.93	1.39	—	—	4.82	1.69	0.838	0.403	—
Language	2.04	0.89	—	—	1.89	1.01	1.797	0.074	—
Abstraction	1.21	0.79	—	—	1.27	0.76	−0.821	0.413	—
Delayed free recall*	1.72	1.37	—	—	2.13	1.46	−3.122	0.002	—
Orientation to time and place	5.29	1.28	—	—	5.41	1.31	−1.002	0.318	—
MMSE	26.14	3.66	—	—	25.97	5.19	0.430	0.668	—

Abbreviations: HAM‐A, Hamilton Anxiety Scale; HAM‐D, Hamilton Depression Scale; MDS‐UPDRS‐III, Part III of the Movement Disorder Society‐Unified Parkinson's Disease Rating Scale; MMSE, Mini‐Mental State Examination; MoCA, Montreal Cognitive Assessment Beijing Version scales.

* Significant improvement at follow‐up.

**FIGURE 1:**
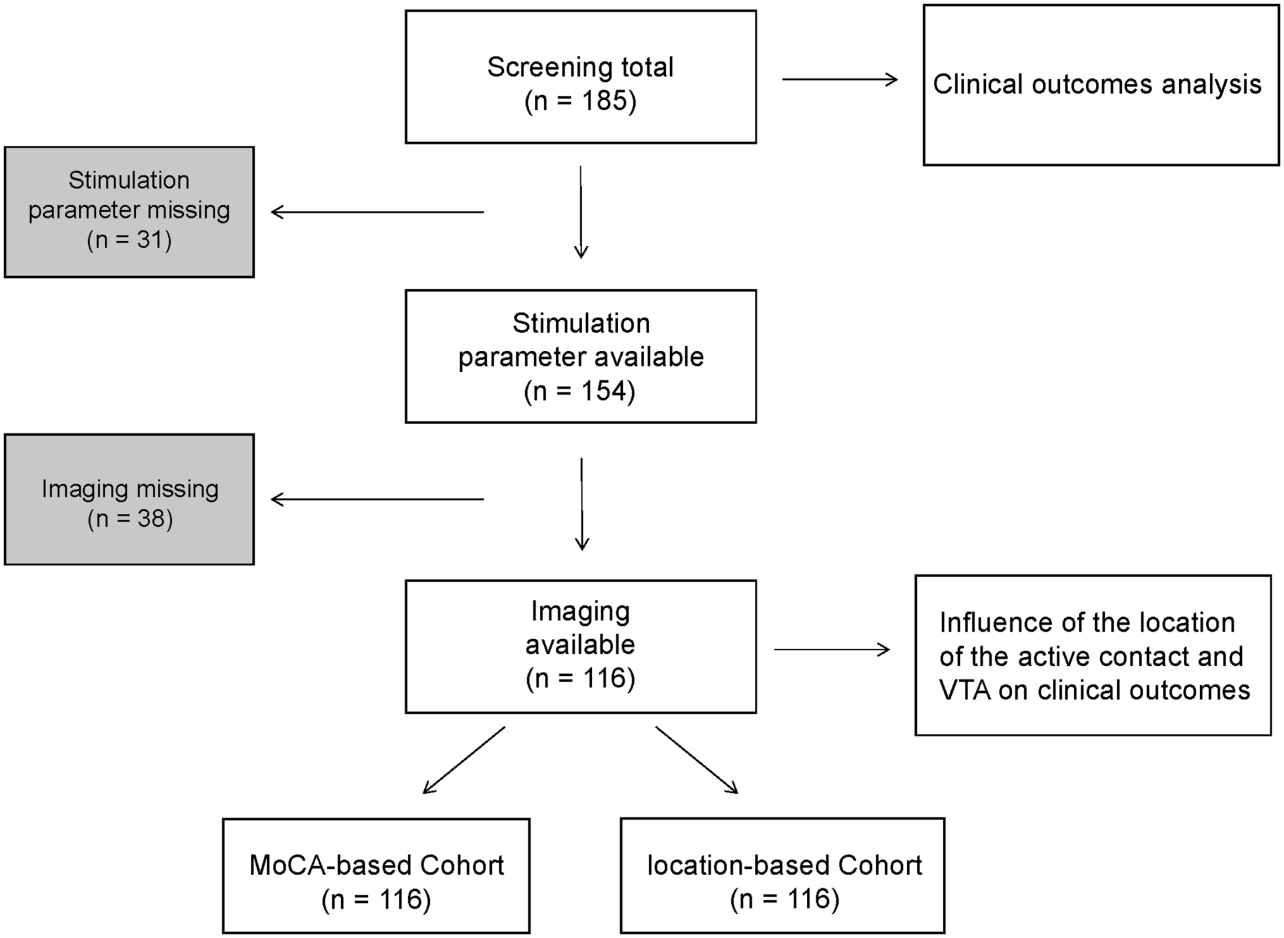
Patient selection and research process.

We further counted the stimulation parameters of the patients at the 12‐month follow‐up. Ultimately, 116 of 185 patients were included. We divided the 116 patients into two cohorts based on the variation of MOCA scores compared to preoperative scores (MoCA‐based cohort) and location of active contacts in the STN (location‐based cohort) (Figure 1).

### 3.2 Influence of the location of active contacts on clinical outcomes

**FIGURE 2:**
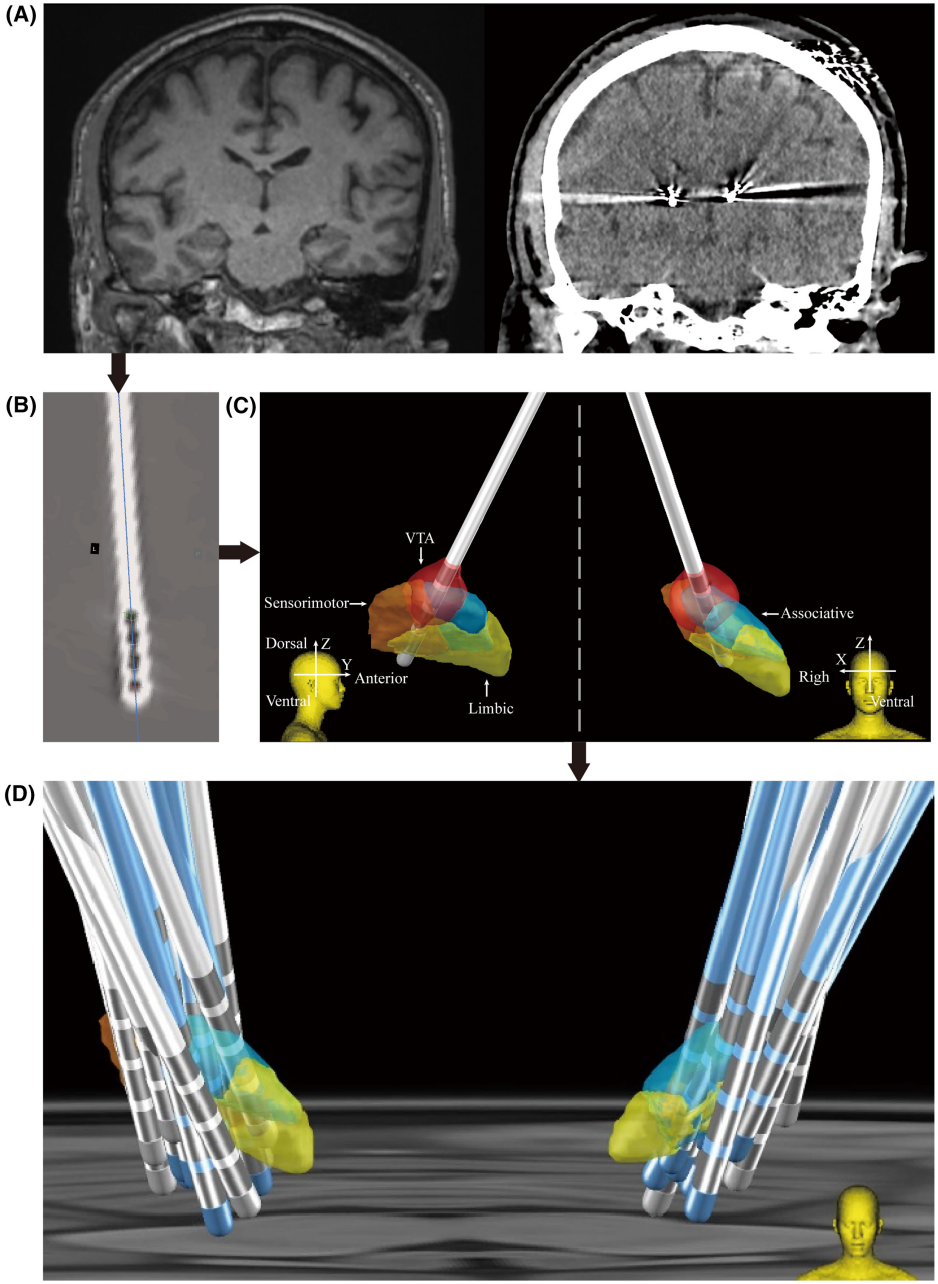
Analysis workflow. (A) Postoperative imaging was co‐registered to preoperative MRI. (B) Normalized to standard space following lead localization. (C) VTAs were calculated. (D) Lead‐Group analysis was used.

In the MoCA‐based cohort, based on the 12‐month follow‐up assessment results of MoCA compared with baseline, patients were divided into the improved group (*n* = 62) and the aggravated group (*n* = 54). Imaging reconstruction was performed by the Lead‐DBS for each patient to determine the lead location and show the active contacts in 3D formats, according to the parameters set at the 12‐month follow‐up. Furthermore, the STN was segmented into three functional zones using DISTAL Minimal Atlas, including the sensorimotor, associative, and limbic zones.^24^ Next, Lead‐Group analysis was used to reconstruct the leads of these patients into MNI standard space, and the spatial position of the active contacts of each patient (marked by X, Y, Z) was counted (Figure 2). Based on the coordinates of the activated contacts, we found that compared to the aggravated group, the improved group had larger values on the Y (*p* < 0.001) and Z (*p* = 0.003) axis of the left hemisphere. Also, group comparisons were made according to the changes in the MMSE scale and the MoCA subscales. The same pattern was found for the MMSE, Visuospatial and Executive abilities, and language ability of MoCA (Table 2).

**TABLE 2:** Mean coordinates of active contacts.

	MoCA		*p*	MMSE		*p*	Visuospatial and Executive abilities		*p*	Language		*p*
	Improved	Aggravated		Improved	Aggravated		Improved	Aggravated		Improved	Aggravated	
X												
R	12.56 ± 1.07	12.60 ± 1.17	0.863	12.53 ± 1.01	12.62 ± 1.23	0.685	12.47 ± 1.08	12.63 ± 1.13	0.495	12.56 ± 1.16	12.76 ± 1.12	0.492
L	−12.22 ± 1.27	−12.26 ± 1.27	0.872	−12.31 ± 1.03	−12.16 ± 1.35	0.517	−12.47 ± 1.16	−12.10 ± 1.19	0.137	−12.26 ± 1.14	−12.41 ± 1.27	0.624
Y												
R	−13.37 ± 1.65	−13.57 ± 1.68	0.559	−13.27 ± 1.56	−13.67 ± 1.76	0.250	−13.19 ± 1.74	−13.62 ± 1.61	0.230	−13.06 ± 1.56	−13.90 ± 1.68	0.044*
L	−12.76 ± 1.67	−13.98 ± 1.52	<0.001*	−12.80 ± 1.47	−13.84 ± 1.81	0.002*	−12.33 ± 1.64	−13.87 ± 1.49	<0.001*	−12.85 ± 1.64	−13.86 ± 1.71	0.020*
Z												
R	−7.41 ± 2.03	−7.62 ± 1.89	0.604	−7.61 ± 1.90	−7.38 ± 2.06	0.570	7.11 ± 2.05	−7.73 ± 1.19	0.134	−7.26 ± 2.05	−7.72 ± 2.12	0.386
L	−6.74 ± 2.25	−7.71 ± 1.74	0.023*	−6.80 ± 1.94	−7.58 ± 2.20	0.070	−6.47 ± 2.35	−7.58 ± 1.82	0.011*	−6.76 ± 2.15	−7.62 ± 1.99	0.108

*Note*: L: left‐STN; R: right‐STN; X, Y, and Z represent the X, Y, and Z axes in MNI standard space.

Abbreviations: MMSE, Mini‐Mental State Examination; MoCA, Montreal Cognitive Assessment Beijing Version scales.

* Significant differences between improved group and aggravated group scores.

It's worth noting that we got similar results in the Location‐based cohort. We reconstructed the lead images of patients without knowing their follow‐up results. We divided the patients into the sensorimotor zone group (*n* = 55) and associative zone group (*n* = 61) based on the location of the active contacts. We found that the mean MoCA score improvement rate at 12 month follow‐up was −2.23% in the sensorimotor zone group, with a deterioration in cognitive function compared to baseline, and 13.56% in the associative zone group, with an improvement in cognitive function (*p* < 0.001). The domain scales of MoCA also showed this trend, such as Visuospatial and Executive abilities (*p* = 0.012), Language ability (*p* < 0.001), and Orientation to time and place ability (*p* = 0.046), indicating that patients with active contacts located in the sensorimotor zone had lower rates of improvement in cognitive symptoms compared to those located in the associative zone. However, the HAM‐A and HAM‐D scores showed opposite results. Patients with active contacts located in the sensorimotor zone showed a better improvement compared with the associative zone (Table 3).

**TABLE 3:** Comparison of symptom improvement rates in different zones of STN.

Scales	Improvement rates % (Mean ± SD)		*p*
	Sensorimotor zone	Associative zone	
MDS‐UPDRS‐III	30.98 ± 70.36	31.17 ± 77.88	0.990
HAM‐A*	49.19 ± 39.34	10.10 ± 55.75	<0.001
HAM‐D*	46.43 ± 43.72	8.12 ± 55.05	<0.001
MMSE	−1.33 ± 16.61	3.84 ± 23.18	0.552
MoCA*	−2.23 ± 16.36	13.56 ± 22.78	<0.001
MoCA domains			
Visuospatial and Executive abilities*	−11.84 ± 70.63	31.79 ± 64.75	0.012
Name	−4.91 ± 35.69	5.48 ± 31.92	0.136
Attention	5.05 ± 33.05	10.94 ± 29.47	0.358
Language*	−12.09 ± 46.78	30.46 ± 59.83	<0.001
Abstraction	1.14 ± 59.54	18.27 ± 66.44	0.190
Delayed free recall	14.61 ± 62.33	39.12 ± 74.07	0.086
Orientation to time and place*	0.23 ± 22.27	9.88 ± 24.56	0.046

Abbreviations: HAM‐A, Hamilton Anxiety Scale; HAM‐D, Hamilton Depression Scale; MDS‐UPDRS‐III, Part III of the Movement Disorder Society‐Unified Parkinson's Disease Rating Scale; MMSE, Mini‐Mental State Examination; MoCA, Montreal Cognitive Assessment Beijing Version scales.

* Significant differences of Improvement rates of scores between Sensorimotor zone and Associative zone.

**FIGURE 3:**
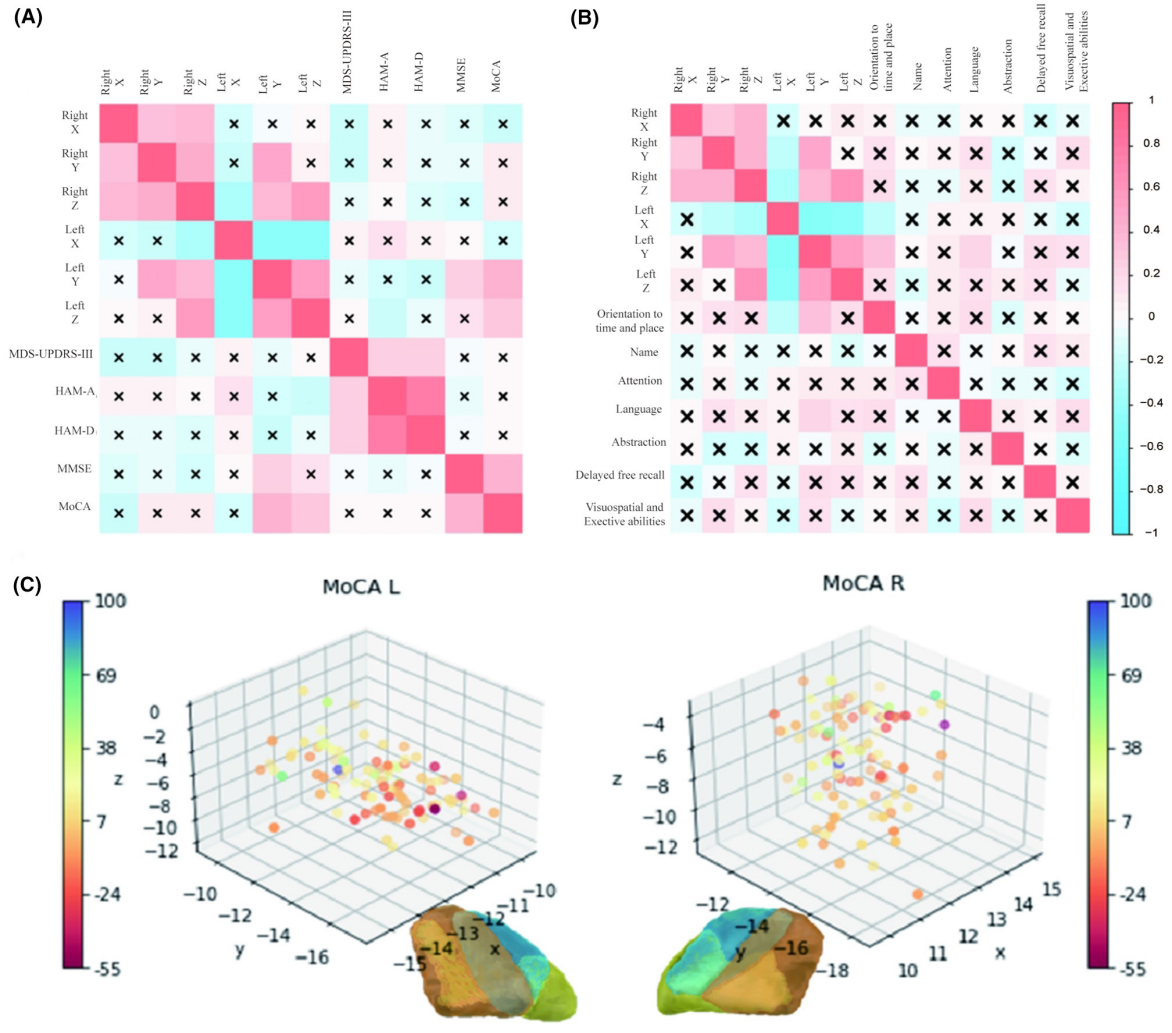
(A) Correlation analysis between coordinate location and symptom improvement rate. (B) Correlation analysis between coordinate location and the improvement of each domain of the MoCA scale. Red represents positive correlation, blue represents negative correlation, and the darker the color, the higher the correlation. X represents *p* < 0.05, which has no statistical significance. (C) Spatial distribution of patients' MoCA improvement rate.

Based on the above findings, we further explored the relationship between the improvement of clinical outcomes of patients and the spatial coordinates of the active contact locations. Therefore, we analyzed the correlation between the coordinate position and the improvement rate of patients' symptoms. The results showed that the improvement rates of MoCA and MMSE were positively correlated with the Y‐value coordinate of the left STN. The improvement rates of MoCA were also positively correlated with the Z‐value coordinate of the left STN. That means, the closer the active contact position of the left cerebral hemisphere was to the anterior side of the STN, the improvement of patients' MoCA and MMSE scores might be better. Similarly, the closer the location of active contacts in the left cerebral hemisphere to the dorsal side of the STN, the better the improvement in MoCA scores, while, for patients' emotional symptoms, we can find that active contacts in the ventral side of the left STN have a positive effect on patients' anxiety symptoms (Figure 3A). Furthermore, in each domain of the MoCA scale, we found that language function and orientation to time and place ability were positively correlated with the value of the left Y‐axis. This implied that stimulation of the anterior side of the STN may have a positive effect on patients' language and orientation to time and place abilities (Figure 3B).

After matching the patient's active contact coordinate points with the patient's MoCA symptom improvement rate, we draw the improvement distribution map in the spatial coordinate, which was used to reflect the coordinate spatial distribution of patients with different improvement rates (Figure 3C).

### 3.3 The effect of VTA on the improvement rate of cognitive symptoms

Based on the parameters of the patients at the 12‐month follow‐up, we calculated the STN–VTA of the patients and counted the overlap of patient‐specific VTA with the sensorimotor, associative, and limbic zones of the STN.

In the MoCA‐based cohort, we also divided the patients into improved and aggravated groups of cognitive function, based on the change in their MOCA scale assessment results at 12 months compared with the assessment results at baseline. We compared the STN–VTA and the VTA of each zone of the STN between these two groups. We found that the overlap of VTA in the sensorimotor (*p* = 0.047) and limbic (*p* = 0.033) zones was significantly smaller in the improved group than in the aggravated group and was mainly reflected in the left hemisphere (*p* = 0.040) (Table 4).

**TABLE 4:** The VTA of different STN and its zones in the MoCA‐based cohort.

STN & STN zones	VTA		*p*
	MoCA improved	MoCA aggravated	
Right hemisphere			
STN	97.96 ± 65.87	110.95 ± 93.482	0.427
Sensorimotor	55.83 ± 46.99	68.67 ± 6.02	0.263
Associative	44.76 ± 34.92	50.50 ± 42.50	0.469
Limbic	28.37 ± 33.83	38.55 ± 40.51	0.181
Left hemisphere			
STN	97.07 ± 65.19	119.71 ± 87.36	0.149
Sensorimotor*	58.48 ± 50.61	82.00 ± 59.79	0.040
Associative	45.50 ± 35.48	49.12 ± 43.33	0.654
Limbic	41.85 ± 33.65	57.95 ± 46.19	0.061
Bihemispheric			
STN	195.11 ± 103.35	230.67 ± 148.32	0.190
Sensorimotor*	114.26 ± 79.07	150.60 ± 97.55	0.047
Associative	90.19 ± 51.65	99.71 ± 72.18	0.453
Limbic*	70.17 ± 52.04	96.64 ± 68.02	0.033

Abbreviations: MoCA, Montreal Cognitive Assessment Beijing Version scales; STN, Subthalamic nucleus; VTA, the volume of tissue activated.

* Significant differences of STN‐VTA between MoCAimproved group and MoCA aggravated group.

### 3.4 The clinical predictive model

**FIGURE 4:**
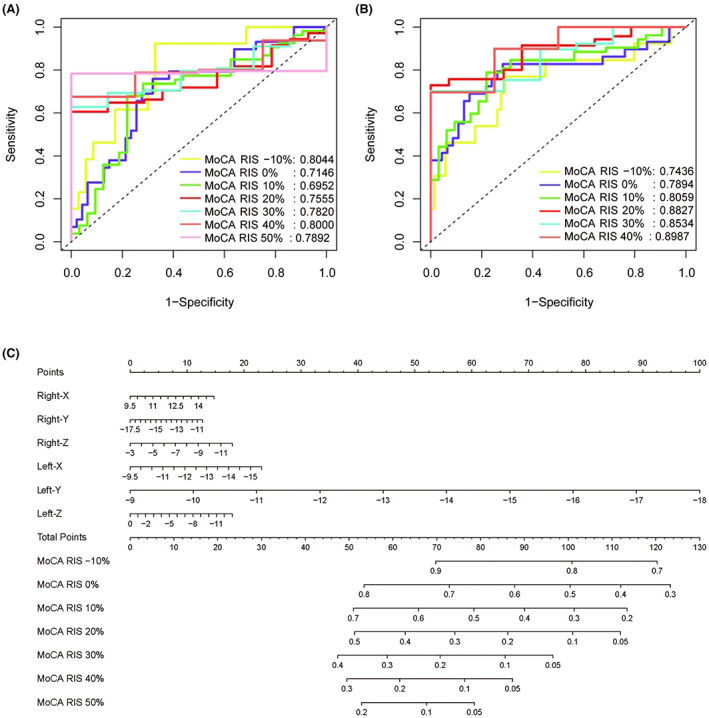
The area under the receiver operating characteristic curve (AUC) and nomogram for the model. (A) The AUC based on coordinate axis information. (B) The AUC based on patient's clinical information. (C) The nomogram based on coordinate axis information. MoCA, Montreal Cognitive Assessment Beijing Version scales; RIS, rate of the improved.

In order to better predict the 12‐month outcome of cognitive and emotional symptoms in PD patients with STN‐DBS, we first constructed a nomogram using Cox regression based on coordinate axes information. The area under the receiver operating characteristic curve (AUC) of the nomogram was 0.8044, 0.7146, 0.6952, 0.7555, 0.7820, 0.8000, and 0.7892 in MoCA −10%, MoCA 0%, MoCA 10%, MoCA 20%, MoCA 30%, MoCA 40%, and MoCA 50%, respectively (Figure 4). Then, we included part of the patient's clinical information in the nomogram, and we found that the AUC of the nomogram was 0.7436, 0.7894, 0.8059, 0.8827, 0.8534, and 0.8987 in MoCA −10%, MoCA 0%, MoCA 10%, MoCA 20%, MoCA 30%, and MoCA 40%, respectively (Figure S1).

## 4 DISCUSSION

In this study, we report the effect of STN‐DBS on clinical symptoms in 185 PD patients followed for 12 months. We found a significant positive effect of STN‐DBS on motor symptoms as well as emotional symptoms for the study. At the 12‐month follow‐up, we also found no significant effect of STN‐DBS on the cognitive function of these patients. However, the active contacts and stimulation parameters used vary from patient to patient. Their clinical improvement also varies widely. So, we further analyzed the effect of the location of the active contacts on the improvement of patients' emotional symptoms and cognitive functions. We found that the effect of STN‐DBS on patients' emotional symptoms and cognitive function was mainly dependent on the spatial location of the active contacts in the STN.

The effect of active contacts location on patients' emotional symptoms and cognitive function is controversial. Eisenstein, In 2014, Eisenstein et al.^25^ suggested that dorsal STN‐DBS could improve patients' emotions. However, in 2017 Gourisankar et al.^26^ proposed that both dorsal and ventral STN‐DBS improved motor function in patients without affecting cognitive function, and ventral STN‐DBS can improve patients' anxiety. Subsequently, Dafsari et al.^27^ in 2018 suggested that anterior, medial, and ventral STN‐DBS were significantly associated with more beneficial non‐motor outcomes. However, the number of cases in the above studies was small and did not consider the stimulation parameters of the patients as well as the generated VTA. One year later, Petry‐Schmelzer et al. in their study proposed that in the dorsal, associative zone of the STN, patients' attention and memory improved more. Emotion symptoms improved with stimulation of the ventral area and sensorimotor zone of the STN.^28^ You et al.^29^ proposed that STN‐DBS not only improves motor symptoms but also cognitive function to a certain extent for PD patients in 2020.

The results of our studies found that on the anterior and dorsal sides of the STN, the patients' MoCA scores improved well. And, on the ventral side of the STN, patients showed good improvement in anxiety symptoms. Similarly, when the active contacts are located in the sensorimotor zone compared to the associative zone, patients have a better emotional improvement rate, but a worse cognitive score improvement rate. Compared to previous studies, we included more patients for clinical outcome analysis (*n* = 185) and analysis of the lead active contact locations (*n* = 116), which is one of the largest study cohort sizes to date. We considered the patients' stimulation parameters and the VTA of each zone of the STN, and we extended our follow‐up to 12 months to make the clinical results more stable, as the parameter settings of the patients tended to be more variable during in 6 months.^30^, ^31^, ^32^

Nomograms have been widely used in clinical work for their personalized application and visualization characteristics. In this study, we added a clinical predictive model based on Cox regression to the study results to predict the clinical outcomes of patients, and we constructed two nomograms: one containing only the electrode axis information and the other incorporating partial clinical features. The visual scoring system can help doctors and patients make more personalized prognostic predictions, which can help them choose better options for further treatment.

Interestingly, during the study, patients' cognitive function changes were more closely related to the left cerebral hemisphere. Some studies have suggested that there are anatomical and functional differences between the left and right cerebral hemispheres. The left cerebral hemisphere is more dominant in language, attentional, and analytical functions.^33^, ^34^, ^35^, ^36^, ^37^, ^38^ Therefore, the differences in the left hemisphere are more significant when cognitive function is assessed. It is also noteworthy that our study showed statistical significance on the MoCA scale scores, while no statistical significance was found on the MMSE scores, although the trends were the same for both. At the same time, previous studies have demonstrated that the MoCA scale is more responsive to the cognitive status of patients compared to the MMSE, so we believe that the MoCA is more trustworthy.^39^, ^40^, ^41^

Some limitations must be considered in our study. First, all assessments were performed during med‐on, and although we counted the medications taken by patients, we did not take into account the variability between medication doses, which may have some impact on the results. Second, the sample size still needs further expansion. We are currently collecting further data for further study.

## 5 CONCLUSION

The results given here in our study provide evidence that the 12‐month outcomes of cognitive and emotional symptoms in PD patients with STN‐DBS were closely related to the specific location of active contacts in the STN and influenced by the VTA.

## FUNDING INFORMATION

This research was supported by grants from the National Natural Science Foundation of China (nos. 81971070) and the Beijing Municipal Administration of Hospitals Clinical Medicine Development of Special Funding Support (grant XMLX201833).

## CONFLICT OF INTEREST STATEMENT

The authors declare that they have no known competing financial interests or personal relationships that could have appeared to influence the work reported in this paper.

## Supporting information


Figure S1

## Data Availability

The data that support the findings of this study are available on request from the corresponding author. The data are not publicly available due to privacy or ethical restrictions.
