# Supplementary figures and images for: Emotional symptoms and cognitive function outcomes of subthalamic stimulation in Parkinson's disease depend on location of active contacts and the volume of tissue activated
Source: CNS Neurosci Ther. 2023 Mar 25;29(8):2355–65. doi: 10.1111/cns.14187 (PMC10352887; doi:10.1111/cns.14187)

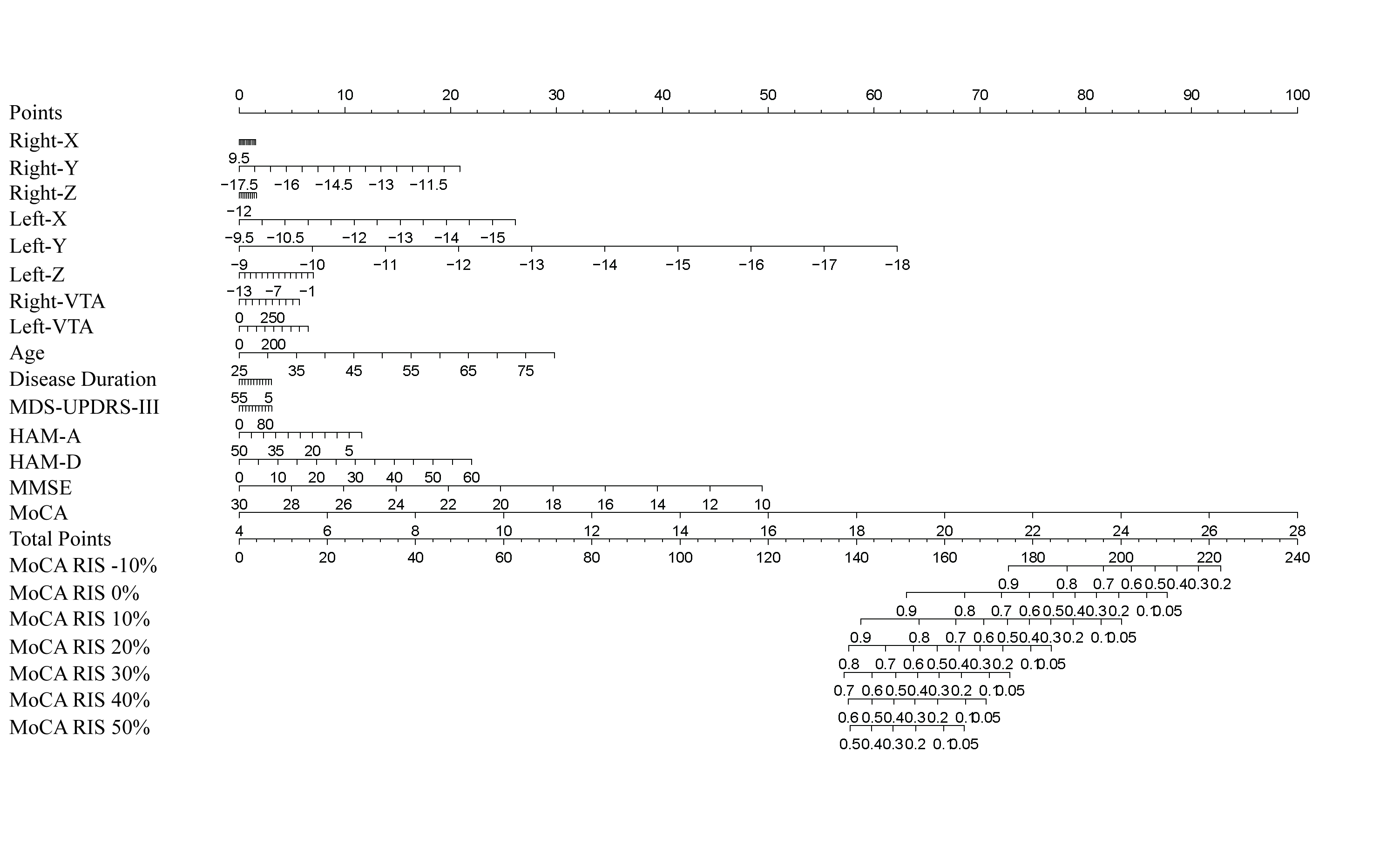

Supplement: Supplementary file 1 — Figure S1 [file CNS-29-2355-s001.tif]
